# Biofluid GPNMB/osteoactivin as a potential biomarker of ageing: A cross-sectional study

**DOI:** 10.1016/j.heliyon.2024.e36574

**Published:** 2024-08-22

**Authors:** Yuan-Yuan Liu, Jing Pang, Chi Zhang, Lv-Tao Zeng, Yao Wang, Shi-Bo Wang, Guo-Qing Fan, Li-Qun Zhang, Tao Shen, Xue-Fei Li, Chuan-Bao Li, Su-Yan Cao, Tie-Mei Zhang, Jian-Ping Cai, Ju Cui

**Affiliations:** a The Key Laboratory of Geriatrics, Beijing Institute of Geriatrics, Institute of Geriatric Medicine, Chinese Academy of Medical Science, Beijing Hospital/National Center of Gerontology of National Health Commission, 1 Dahua Rd, Dongcheng District, Beijing, PR China; b Department of Rheumatology, Beijing Hospital, National Center of Gerontology, Institute of Geriatric Medicine, Chinese Academy of Medical Sciences, PR China; c Shenzhen Institute of Synthetic Biology, Shenzhen Institute of Advanced Technology, Chinese Academy of Sciences, Shenzhen, PR China; d Department of Laboratory Medicine, Beijing Hospital, National Center of Gerontology, Institute of Geriatric Medicine, Chinese Academy of Medical Sciences, PR China; e Department of General Practice/VIP Medical Service, Beijing Hospital, National Center of Gerontology, Institute of Geriatric Medicine, Chinese Academy of Medical Sciences, PR China

**Keywords:** GPNMB, Biomarker, Aging, Urine, Serum

## Abstract

**Background:**

Glycoprotein non-metastatic melanoma B (GPNMB)/osteoactivin was first identified in the human melanoma cell lines. GPNMB plays a key role in the anti-inflammatory and antioxidative functions as well as osteoblast differentiation, cancer progression, and tissue regeneration. Recently, GPNMB was used as an anti-aging vaccine for mice. The present study aimed to investigate the potential of biofluid GPNMB as an aging biomarker in humans using serum and urine samples from an aging Chinese population.

**Methods:**

We analyzed RNA-sequencing data (GSE132040) from 17 murine organs across different ages to assess the gene expression of potential ageing biomarkers. Spearman's correlation coefficients were used to evaluate the relationship between gene expression and age. Meanwhile, a cross-sectional population study was conducted, which included 473 participants (aged 25–91 years), a representative subset of participants from the Peng Zu Study on Healthy Ageing in China (Peng Zu Cohort). Biofluid GPNMB levels were measured by ELISA. The associations of serum and urine GPNMB levels with various clinical and anthropometrical indices were assessed using ANOVA, Kruskal-Wallis H test, and univariate and multivariate linear regression analyses.

**Results:**

In mice, the *Gpnmb* mRNA expression levels showed a significant positive association with age in multiple organs in mice (P < 0.05). In Peng Zu Cohort, biofluid (both serum and urine) GPNMB levels showed a positive correlation with age (P < 0.05). Univariate linear regression analysis revealed that serum GPNMB levels were negatively associated with skeletal muscle mass index (SMI, P < 0.05) and insulin-like growth factor 1 (IGF-1, P < 0.05), and urine GPNMB levels showed a negative association with total bile acids (TBA, P < 0.05). Multivariate linear regression analysis further indicated that serum GPNMB levels negatively correlated with the systemic immune-inflammation index (SII, P < 0.05), and the urine GPNMB levels maintained a negative association with TBA (P < 0.05), additionally, urine GPNMB levels in men were significantly lower than in women (P < 0.05).

**Conclusions:**

The biofluid GPNMB was a strong clinical biomarker candidate for estimating biological aging.

## 1 Background

The aging global population has significant adverse effects on the social and economic development globally. According to the World Health Organization estimates, 2.1 billion people are expected to be above 60 years of age by the year 2050, and 426 million people are expected to be 80 years and above [1]. Therefore, the concept of healthy aging has been proposed so that the older individuals could maintain functional ability for as long as possible. Aging is a significant risk factor for numerous chronic diseases. Therefore, the discovery of aging biomarkers is important for the health monitoring of older subjects. This can have a positive impact on healthy aging and also increase the understanding of the biological aging process.

Proteins in biofluids such as blood and urine can serve as biomarkers for aging and related diseases. Across the lifespan of an individual, distinct alterations in the blood protein levels modulate distinct biological pathways [2,3]. The infusion of blood from young mice reversed the effects of aging and related diseases in the older mice [4]. This suggested that monitoring the molecular changes in blood would give a better understanding of the aging process. The urinary protein profile also reflects the metabolic and pathophysiological status of an individual. Analysis of the urinary protein profiles of elderly subjects demonstrated a decline in the renewal of the extracellular matrix and the immune functions, and were associated with the impairments in cardiovascular tissue remodeling and immune function [5]. The changes were distinctly observed in collagen, uromodulin, and fibrinogen [6].

Weterman et al. first reported the expression of glycoprotein non-metastatic melanoma B (*GPNMB*) in the low-metastatic human melanoma cell lines [7]. Safadi et al. analyzed the osteopetrosis rat model and reported that osteoactivin, the rat homolog of GPNMB, played an active role in the biosynthesis of the bone matrix and its mineralization [8]. GPNMB is a type I transmembrane protein that consists of a signal peptide, an extracellular domain, a single-pass transmembrane domain, and a cytoplasmic domain [9]. The extracellular domain of GPNMB can be cleaved by sheddases to generate a soluble extracellular fragment with signaling ability in the stromal cells [10,11]. GPNMB is widely expressed in several cells and tissues under normal conditions, and significantly elevated in several malignant tissues including glioma, melanoma, breast cancer cells, etc [12,13].

Suda et al. analyzed the transcriptome data from senescent human vascular endothelial cells and identified the transmembrane GPNMB protein as a candidate seno-antigen [14]. They demonstrated that vaccination against GPNMB in the male premature ageing model mice extended their lifespan by 5.4 weeks, mainly eliminated the *Gpnmb*-positive senescent cells, and attenuated age-related pathologies such as atherosclerosis and metabolic abnormalities [14]. In the senescent cells, GPNMB maintained lysosomal integrity as a survival factor, the overexpression of *Gpnmb* in the adipocytes, endothelial cells, and macrophages protected mice from stress-induced premature senescence and attenuated dietary vascular dysfunction and atherogenesis [15].

The tight control of GPNMB-targeting vaccine for humans is necessary. However, as a seno-antigen candidate, GPNMB is a promising aging biomarker in humans. In this study, we obtained serum and urine samples from 473 Chinese residents (226 males and 247 females; aged 25–91 years) that were randomly selected from the Peng Zu Study on Healthy Aging in China (Peng Zu Cohort). Then, we analyzed the relationship of biofluid GPNMB levels with human aging by determining the association between biofluid GPNMB levels and various physical indices of the study cohort.

## 2 Methods

1. Data collection

GSE132040 dataset was downloaded from the Gene Expression Omnibus (GEO) database in the NCBI (https://www.ncbi.nlm.nih.gov/geo/) website. This dataset contains the transcriptomic information of 17 organs from *Mus musculus* across the organism's life span.

2. Study population

We included 473 participants (226 males and 247 females) that were randomly selected from the Peng Zu Cohort [16], and consisted of 146 young subjects (≤40 years old), 153 middle-aged subjects (41–60 years old), 98 young-old subjects (61–74 years old), and 76 old-old subjects (≥75 years old) adults. All the study participants were recruited from the health examination centers and communities in Beijing through advertisements in 2020. The eligibility criteria were as follows: (1) age ≥25 years; (2) signed informed consent form; (3) no history of alcohol or drug abuse; and (4) no history of mental disorders. The exclusion criteria were as follows: (1) acute medical treatment or hospitalization within the first 3 months of measurement; (2) presence of severe diseases such as acute heart/liver/kidney disease and respiratory failure; and (3) inability to walk independently.

3. Clinical measurements

Anthropometrical measurements, including height, weight, body mass index (BMI), appendicular skeletal muscle mass, and total body fat mass were obtained for all the included study subjects. The height and weight of the subjects were measured with a digital scale. BMI was calculated as the weight of the subject (in kgs) divided by the square of height (in meters). Appendicular skeletal muscle mass and total body fat mass were estimated using the bioelectrical impedance analysis (BIA) instrument (BCA-2A; Tsinghua Tong Fang Co. Ltd, Beijing China). The body fat percentage (BFP) was calculated as the total body fat mass divided by the total body weight, and multiplied by 100. Skeletal muscle mass index (SMI) was calculated as the appendicular skeletal muscle mass divided by the BMI [17].

4. Serum and urine sample preparation and biomedical measurements

The blood samples were collected from the participants after overnight fasting (∼12 h) in chilled serum tubes and K2 EDTA vacutainer tubes. The samples were left at room temperature for 20 min to allow the clotting of blood. Then, the samples were centrifuged at 1000×*g* for 10 min and the separated serum was harvested and stored in a refrigerator at −80 °C for further assays. The serum samples were used to assess the concentrations of hypersensitive C-reactive protein (hs-CRP), alanine aminotransferase (ALT), aspartate aminotransferase (AST), glutamyl transpeptidase (GGT), total bile acid (TBA), blood creatinine (CRE), blood uric acid (URIC), blood urea (UREA), fasting blood glucose (FBG), total cholesterol (TC), triglyceride (TG), high-density lipoprotein-C (HDL-C), low density lipoprotein-C (LDL-C), and non-esterified fatty acid (NEFA) using the Hitachi Automatic Analyzer (LABOSPECT 008 AS, Japan). The insulin-like growth factor 1 (IGF-1) levels were estimated using a solid-phase enzyme-linked chemiluminescent immunoassay with an Immulite 2000 system (Siemens Healthcare Diagnostics, Inc). The β-galactosidase activity in serum was measured using a β-galactosidase activity assay kit (Elabscience Biotechnology Co., Ltd, China) via the colorimetric method. To evaluate hematology parameters, the blood samples in the K2 EDTA tubes were completely mixed with the additive by inversion. The tubes were then immediately stored at 4 °C and the hematology parameters were evaluated within 24 h. The blood counts of white blood cells (WBCs), red blood cells (RBCs), hemoglobin (Hb), platelets, neutrophils, lymphocytes, monocytes, eosinophils, and basophils were measured using an automatic hematology analyzer (Sysmex XN-20 system, Japan). The percentage of eosinophils (EO %) and basophils (BASO %) were calculated automatically.

The midstream specimen of the first-morning urine was collected in a sterile container and centrifuged at 500×*g* for 5–10 min at 4 °C. The supernatants were collected and stored in a refrigerator at −80 °C. The urine creatinine levels were assessed using the Hitachi Automatic Analyzer (LABOSPECT 008 AS, Japan). The urine osmotic pressure was measured using an automatic cryoscopic osmometer (Gonotec OSMOMAT 030, Germany).

The neutrophil-to-lymphocyte ratio (NLR), platelet-to-lymphocyte ratio (PLR), and lymphocyte-to-monocyte ratio (LMR) were estimated by dividing the number of neutrophils, platelets, or lymphocytes by the number of lymphocytes or monocytes. The systemic immune-inflammation index (SII) was calculated as the ratio of the neutrophil counts to the lymphocyte counts multiplied by the platelet counts [18]. The estimated glomerular filtration rate (eGFR) was calculated using the following full age spectrum (FAS) equation: FAS−eGFR=107.3/(Scr/QScr) for those aged 2–40 years or ${\text{FAS}-\text{eGFR}=\hat{0.988}}^{\left(\text{age}-40\right)}\times 107.3/\left(\text{Scr}/\text{QScr}\right)$ for those aged >40 years, where Scr = serum creatinine and QScr = 7 mg/L for females and 9 mg/L for males [19].

The serum and urine GPNMB concentrations were measured using the ELH-Osteoactivin enzyme-linked immunosorbent assay (ELISA) kit (RayBiotech, Inc, Norcross, GA, USA) according to the manufacturer's instructions. The assay detection range was 49.15–12000 pg/mL. The intra-assay coefficient of variation (CoV) was less than 10 % and the inter-assay CoV was less than 12 %. The serum samples were diluted 10 times, whereas the urine samples were not diluted.

5. Statistical analysis

Statistical analysis was performed using the R statistical software (Version 4.1.3) and the IBM SPSS software version 26.0 (IBM, Armonk, NY, USA). Based on the normality of the distribution, continuous variables were expressed as mean ± standard deviation (SD) or median (25 %, 75 %). For the Gaussian distributed data, differences between groups were analyzed using the one-way analysis of variance (ANOVA), followed by Dunnett's test for multiple comparisons. For the non-normally distributed data, differences between groups were analyzed using the Kruskal-Wallis H test followed by a Bonferroni correction. The correlation analysis was performed to estimate the Pearson's or Spearman's correlation coefficients for the relationships between biofluid GPNMB levels and various parameters. To compare the trends of the biofluid GPNMB levels, GPNMB concentrations were converted to a Z-score by subtracting the mean from each concentration and divided by the SD. Univariate and multivariate linear regression analyses were performed to determine the association of the biofluid GPNMB levels with age and other variables. Two-sided P < 0.05 was considered statistically significant.

## 3 Results

1. Elevated *Gpnmb* mRNA expression is elevated in 17 murine organs across the lifespan

Adipose, muscle, and bone tissues secrete distinct families of cytokines known as adipokines, myokines, and osteokines, respectively, which are closely associated with chronic low-grade inflammation and a variety of age- and metabolism-related diseases [20]. Therefore, these cytokines are promising biomarker candidates for estimating biological aging. To examine if the expression levels of these cytokines were associated with aging in different organs or tissues, we analyzed a readily available dataset (GSE132040) that included bulk RNA-sequencing data for 17 murine organs at 1, 3, 6, 9, 12, 15, 18, 21, 24, and 27 months [21]. Specifically, we analyzed adipokine genes including *Retn, Adipoq, Wisp1, Rarres2, Sparc, Sfrp5, Nampt, Fstl1, Ahsg, Lcn2, Nucb2, Fgf21* and *Grn*; myokine genes including *Fndc5, Dcn, Mstn, Il15, Igf1, Bdnf, Lif, Tnf* and *Il6*; and osteokine genes *Sost* and *Gpnmb* [22,23]. This comprehensive analysis aims to explore representative markers in biological aging.

**Fig. 1:**
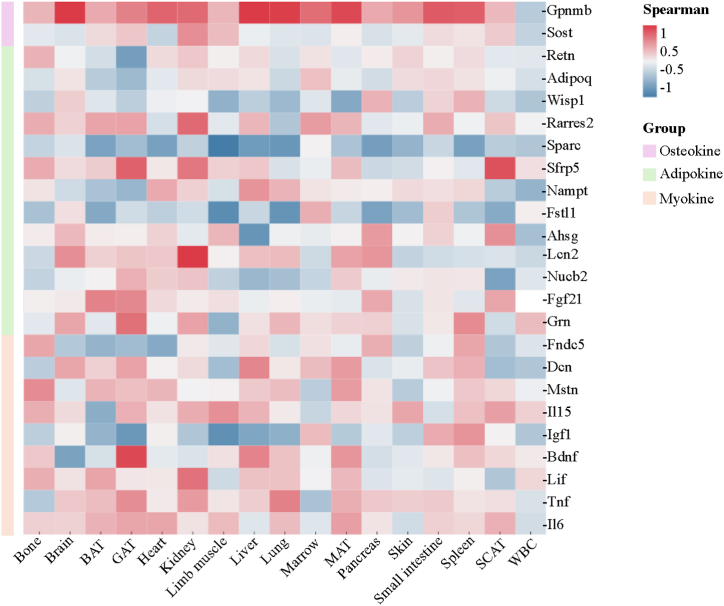
**Gpnmb expression levels are highly correlated with age in multiple mouse tissues.** Spearman correlation coefficients between gene expression levels and age in 17 tissues across mice lifespan. Adipokine genes (*Retn, Adipoq, Wisp1, Rarres2, Sparc, Sfrp5, Nampt, Fstl1, Ahsg, Lcn2, Nucb2, Fgf21, Grn*), myokine genes (*Fndc5, Dcn, Mstn, Il15, Igf1, Bdnf, Lif, Tnf, Il6*), osteokine genes (*Sost, Gpnmb*) were analyzed, and the *Gpnmb* expression levels are highly correlated with age in multiple mouse tissues. The raw data is from bulk RNA-sequencing of 17 organs and plasma proteomics at 10 ages (aged 1, 3, 6, 9, 12, 15, 18, 21, 24, 27 months) (GSE132040). BAT, brown adipose tissue; GAT, gonadal adipose tissue; MAT, mesenteric adipose tissue; SCAT, subcutaneous adipose tissue; WBC, white blood cell. (For interpretation of the references to color in this figure legend, the reader is referred to the Web version of this article.)

Fig. 1 shows the Spearman's correlation coefficients for the relationship between age and the expression levels of these adipokine, myokine, and osteokine genes in 17 murine tissues such as bone, brain, brown adipose tissue (BAT), gonadal adipose tissue (GAT), heart, kidney, limb muscle, liver, lung, marrow, mesenteric adipose tissue (MAT), pancreas, skin, small intestine, spleen, subcutaneous adipose tissue (SCAT), and WBCs. Among all the cytokine genes analyzed, *Gpnmb* showed the highest correlation with age in multiple mouse tissues. The expression levels of *Gpnmb* showed significant positive association with age in the brain (r = 0.680, p < 0.001), GAT (r = 0.477, p < 0.001), heart (r = 0.583, p < 0.001), liver (r = 0.748, p < 0.001), lung (r = 0.611, p < 0.001), marrow (r = 0.560, p < 0.001), MAT (r = 0.460, p < 0.001), spleen (r = 0.490, p < 0.001), pancreas (r = 0.434, p = 0.001), small intestine (r = 0.424, p = 0.001), bone (r = 0.298, p = 0.027), kidney (r = 0.314, p = 0.019), and skin (r = 0.295, p = 0.036). It is worth noting that *Sparc* exhibits a significant negative correlation with age across various mouse organs. *Gpnmb* and *Sparc* are both bone-related genes. SPARC, also known as osteonectin, is a widely expressed profibrotic secreted protein with pleiotropic roles [24]. Negative correlation of *Sparc* with age across tissue types suggests that *Sparc* may also be a potential biomarker of aging, as well as a target for anti-aging treatment, which warrants further investigation. The results of the Spearman correlation analysis between the expression levels of all the selected cytokine genes and age in the 17 murine organs are shown in Table S1.

**Fig. 2:**
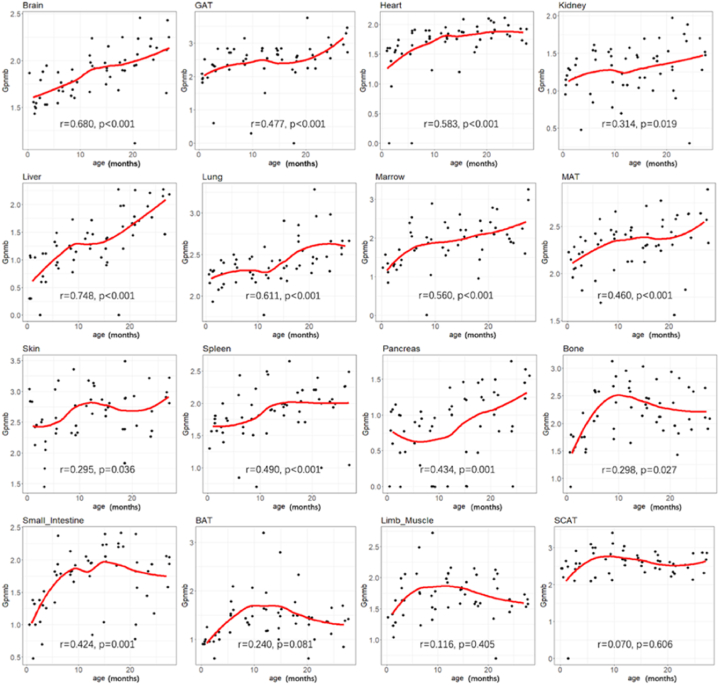
**Expression of *Gpnmb* gene in 16 organs across mice lifespan by high throughput sequencing.** The trend graph of *Gpnmb* expression levels with age and the Spearman correlation coefficients between the *Gpnmb* expression levels and age were indicated in the 16 organs of mice. BAT, brown adipose tissue; GAT, gonadal adipose tissue; MAT, mesenteric adipose tissue; SCAT, subcutaneous adipose tissue. (For interpretation of the references to color in this figure legend, the reader is referred to the Web version of this article.)

The mRNA levels of *Gpnmb* in 16 organs across the mouse lifespan were further analyzed. Since *Gpnmb* mRNA was not detected in many WBC samples, it was excluded from the analyses. As shown in Fig. 2, *Gpnmb* mRNA levels increased with age in most of the organs and peaked at 12 months. Furthermore, after 18 months, *Gpnmb* mRNA levels continued to significantly increase with age in 11 organs (brain, GAT, heart, kidney, liver, lung, marrow, MAT, skin, spleen, and pancreas), but *Gpnmb* mRNA levels decreased slightly in the bones and the small intestine after 12 months. Collectively, the expression levels of *Gpnmb* were higher in most organs and tissues of older mice compared to those in younger mice. These findings suggest that GPNMB levels in human may increase with aging. To explore this possibility, we detected the GPNMB levels in the biofluids of human subjects across different age groups. It is important to note that the GEO dataset analysis in mice focused on the bulk RNA-sequencing data, reflecting the gene expression levels of *Gpnmb* with age. GPNMB in human biofluids is the soluble form, requiring cleavage by sheddases to be released into circulation. We supposed the gene expression levels observed in tissue could correlate with the levels of soluble GPNMB in biofluids, as increased gene expression may lead to higher availability of the protein for shedding and subsequent release into the bloodstream and urine.

2. Baseline characteristics of study subjects

The cohort data stratified by chronological age is shown in Table 1. According to chronological age, the study participants were divided into the following four groups: young (≤40 years old), middle-aged (41–60 years old), young-old (61–74 years old), and old-old (≥75 years old). The age-related changes in the anthropometrical characteristics, complete blood counts, liver and kidney functional indices, glycolipid metabolism, and serum IGF-1 levels were subsequently analyzed between these groups.

**Table 1:** Baseline characteristics stratified by age.

Characteristics	Young (≤40)	Middle-aged (41–60)	Young-old (61–74)	Old-old (≥75)	P
	n = 146	n = 153	n = 98	n = 76	
Age (years)	30.50 (27.00, 34.00)	51.00 (48.00, 55.50)	67.00 (65.00, 71.00)	80.50 (78.00, 83.75)	–
Sex (male, %)	52 (35.60 %)	70 (45.80 %)	61 (62.20 %)	43 (56.60 %)	–
**Anthropometrical characteristics (including somatotype and body composition)**					
BMI	22.94 ± 3.40	24.43 ± 3.27	24.55 ± 2.88	25.92 ± 3.04	< **0.001**
BFP	24.87 ± 6.13	26.43 ± 5.38	27.26 ± 4.49	33.42 ± 4.49	< **0.001**
SMI	0.99 ± 0.17	0.94 ± 0.14	0.93 ± 0.11	0.79 ± 0.18	**0.008**
**Complete blood count and relevant indicators**					
WBC (10^9/L)	6.00 ± 1.39	5.71 ± 1.45	6.19 ± 1.41	6.91 ± 3.04	**0.038**
RBC (10^12/L)	4.69 ± 0.44	4.68 ± 0.44	4.82 ± 0.44	4.53 ± 0.52	**0.024**
Hb (g/L)	139.85 ± 13.54	140.87 ± 14.38	147.98 ± 12.54	136.84 ± 14.57	**0.001**
EO%	1.60 (1.00, 2.85)	1.90 (1.20, 3.30)	1.55 (1.00, 2.63)	1.80 (1.30, 2.80)	0.435
BASO%	0.57 ± 0.30	0.70 ± 0.31	0.66 ± 0.26	0.56 ± 0.25	< **0.001**
NLR	1.56 (1.25, 1.94)	1.65 (1.28, 2.09)	1.74 (1.17, 2.49)	1.67 (1.32, 2.61)	0.134
PLR	126.33 ± 37.13	139.25 ± 51.08	124.95 ± 48.04	120.79 ± 59.77	**0.044**
LMR	6.73 ± 2.14	6.16 ± 1.99	5.52 ± 1.84	5.19 ± 1.67	< **0.001**
SII	400.68 (292.99, 519.89)	408.84 (297.13, 549.38)	416.43 (249.94, 581.25)	403.92 (297.60, 482.74)	0.959
Hs-CRP (mg/L)	0.57 (0.32, 1.37)	0.74 (0.37, 1.46)	0.81 (0.43, 1.49)	1.02 (0.60, 2.88)	0.112
**Liver function**					
ALT (U/L)	15.00 (11.00, 22.30)	18.50 (15.00, 26.00)	19.00 (15.00, 25.00)	16.50 (14.00, 24.00)	**0.005**
AST (U/L)	18.00 (15.00, 21.00)	19.50 (17.00, 23.00)	19.00 (17.00, 23.00)	20.50 (18.00, 25.75)	< **0.001**
GGT (U/L)	14.00 (11.00, 22.00)	20.00 (14.00, 31.00)	21.00 (15.00, 29.00)	22.00 (16.00, 28.00)	< **0.001**
TBA (μmol/L)	3.10 (2.00, 4.30)	2.50 (1.60, 3.80)	3.20 (1.90, 5.10)	3.00 (1.60, 5.90)	**0.034**
**Renal function**					
CRE (μmol/L)	60.00 (52.00, 72.00)	65.00 (55.25, 76.75)	72.00 (60.00, 82.00)	73.00 (61.00, 86.00)	< **0.001**
URIC (μmol/L)	290.00 (239.75, 351.00)	307.50 (249.75, 365.75)	322.00 (274.00, 379.00)	322.50 (288.00, 385.25)	**0.002**
UREA (mmol/L)	5.17 (4.26, 5.99)	5.11 (4.26, 6.06)	5.48 (4.74, 6.38)	6.50 (5.27, 7.38)	< **0.001**
FAS-eGFR	117.75 (107.00, 130.48)	100.86 (90.04, 112.28)	80.88 (69.90, 88.96)	66.50 (53.02, 74.52)	< **0.001**
**Blood glucose and blood lipids**					
FBG (mmol/L)	5.10 (4.80, 5.30)	5.40 (5.00, 5.80)	5.60 (5.10, 7.30)	6.00 (5.50, 7.07)	< **0.001**
TC (mmol/L)	4.51 ± 0.74	5.05 ± 0.90	4.89 ± 0.96	4.48 ± 1.05	< **0.001**
TG (mmol/L)	0.71 (0.50, 1.02)	1.03 (0.75, 1.68)	1.31 (0.96, 1.72)	1.16 (0.89, 1.49)	< **0.001**
HDL-C (mmol/L)	1.45 ± 0.32	1.44 ± 0.35	1.37 ± 0.40	1.36 ± 0.28	0.088
LDL-C (mmol/L)	2.66 ± 0.68	3.16 ± 0.82	2.92 ± 0.89	2.61 ± 0.93	< **0.001**
NEFA (mmol/L)	0.38 ± 0.17	0.45 ± 0.18	0.53 ± 0.22	0.64 ± 0.22	< **0.001**
**Other**					
β-galactosidase (U/L)	84.20 ± 13.00	95.61 ± 67.12	103.58 ± 14.91	111.97 ± 23.58	**0.012**
IGF-1 (ng/mL)	207.34 ± 46.43	155.36 ± 43.25	133.23 ± 34.33	104.10 ± 48.12	< **0.001**
Urine osmotic pressure	0.86 (0.65, 1.01)	0.79 (0.64, 0.92)	0.71 (0.53, 0.85)	0.56 (0.45, 0.69)	< **0.001**
Urine creatinine (μmol/L)	12861.00 (8697.00, 19330.00)	12269.00 (8026.00, 16897.00)	13214.00 (8077.00, 15950.00)	7908.00 (5052.00, 13196.00)	< **0.001**
Serum GPNMB conc. (pg/mL)	10428.26 ± 6379.71^b^	11653.16 ± 7238.96	13589.28 ± 10341.66^b^	11872.50 ± 11970.22	**0.038**
Urine GPNMB conc. (pg/mL)^a^	240.67 (120.84, 354.53)^b^	282.38 (153.86, 432.03)^b^	271.58 (171.85, 488.34)	259.93 (124.94, 448.71)	**0.022**

Data are presented as the mean value ± SD or median (percent 25, percent 75). Differences among groups were analyzed by the one-way ANOVA and Kruskal-Wallis H test. Bold means P < 0.05. ALT, alanine aminotransferase; AST, aspartate aminotransferase; BASO%, basophil percentage; BFP, body fat percentage; BMI, body mass index; CRE, blood creatinine; EO%, eosinophil percentage; FAS-eGFR, estimated glomerular filtration rate (calculated as the FAS equation); GGT, glutamyl transpeptidase; Hb, hemoglobin; HDL-C, high density lipoprotein-C; Hs-CRP, hypersensitive C-reactive protein; IGF-1, insulin-like growth factor 1; LDL-C, low density lipoprotein-C; LMR, lymphocyte-to-monocyte ratio; NEFA, non-esterified fatty acid; NLR, neutrophil-to-lymphocyte ratio; PLR, platelet-to-lymphocyte ratio; RBC, red blood cell; SII, systemic immune-inflammation index; SMI, skeletal muscle mass index; TBA, total bile acid; TC, total cholesterol; TG, triglyceride; UREA, blood urea; URIC, blood uric acid; WBC, white blood cell.

a Analyzed as logarithmically transformed (log10 scale).

b P < 0.05 in post-hoc test.

The anthropometrical characteristics, including somatotype and body composition, BMI (P < 0.001) and BFP (P < 0.001) increased with age, whereas SMI (P = 0.008) decreased with age. Furthermore, the number of WBCs and RBCs, Hb level, BASO%, PLR, and LMR varied with age. The LMR was significantly higher in the young group compared with the other groups (P < 0.001). The number of RBCs (P = 0.024) and Hb level (P = 0.001) were significantly higher in the young-old group compared with the other groups. The number of WBCs (P = 0.038) was highest in the old-old group compared with the other groups. BASO% (P < 0.001) and PLR (P = 0.044) were significantly higher in the middle-aged group compared with the other groups. Most of the liver function indices in the serum were significantly increased with age. The young-old group showed the highest serum levels of ALT (P = 0.005) and TBA (P = 0.034), whereas the serum levels of AST (P < 0.001) and GGT (P < 0.001) were highest in the old-old group. Regarding the kidney function indices, the levels of CRE (P < 0.001), URIC (P = 0.002), and UREA (P < 0.001) increased with age. However, FAS-eGFR (P < 0.001) decreased significantly with age, including a decrease of 40 % in the FAS-eGFR in subjects ≥75 years. Moreover, blood glucose and lipid levels also showed significant changes in different age groups. The levels of FBG (P < 0.001) and NEFA (P < 0.001) significantly increased with age. The levels of TG (P < 0.001) were highest in the young-old group. The levels of TC (P < 0.001) and LDL-C (P < 0.001) were the highest in the middle-aged group. The urine creatinine levels (P < 0.001), and urine osmotic pressure (P < 0.001) were lowest in the old-old group.

The serum IGF-1 levels (P < 0.001) reduced progressively with age, and were consistent with reports from previous studies [25,26]. IGF-1 is a homeostatic regulator of several physiological, anabolic, and metabolic processes and plays a key role in growth, development, and ageing [27]. After reaching adulthood, IGF-1 secretion declined continuously to very low levels [26]. Concurrently, serum β-galactosidase activity was measured to evaluate aging in this cohort, showing a significant positive correlation with age (P = 0.012), consistent with previous studies [28], β-galactosidase is the most widely used biomarker for senescent cells, tissues [15], and biofluids [28], further validating our cohort.

3. Biofluid GPNMB levels show positive correlation with age

In this study, we focused on GPNMB levels in the body fluids of different age groups using ELISA. GPNMB levels in both serum (P = 0.038) and urine (P = 0.022) samples showed a significant correlation with age. In further post-hoc test, serum GPNMB concentrations were significantly higher in the young-old group (61–74 years old) compared to the young group (≤40 years old)(P = 0.045). And urine GPNMB concentrations were significantly higher in the middle-aged group (41–60 years old) compared to the young group (P = 0.027). The post-hoc tests improve the reliability of the results, but this may increase the risk of false negatives (i.e., failing to detect actual significance). To address this, we also performed Pearson's correlation analyses to further assess the relationship between age and the GPNMB levels in the biofluids.

**Fig. 3:**
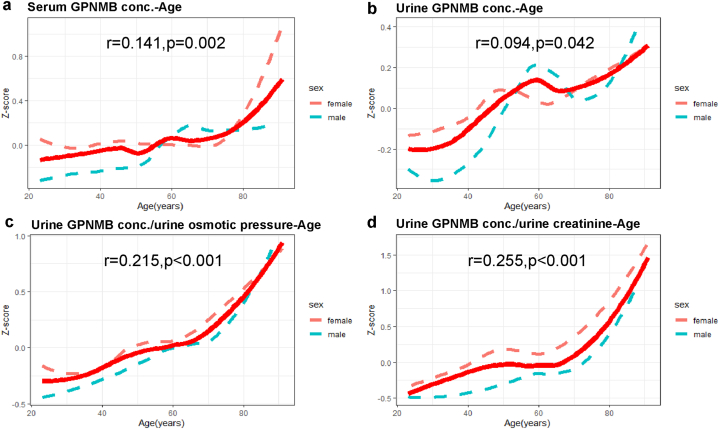
**Correlations between age and biofluid GPNMB concentration.** (a) The correlation between age and serum GPNMB concentration. (b) The correlation between age and urine GPNMB concentration. (c)The correlation between age and urine GPNMB concentration normalized by urine osmotic pressure. (d) The correlation between age and urine GPNMB concentration normalized by urine creatinine. GPNMB concentrations were scaled (converted to Z values) by subtracting the sample mean from individual concentrations and dividing by the standard deviation.

Serum GPNMB levels showed a positive correlation with age (r = 0.141, p = 0.002) (Fig. 3A). Urine GPNMB levels (r = 0.094, p = 0.042), urine GPNMB levels normalized by osmotic pressure (r = 0.215, p < 0.001), and urine GPNMB levels normalized by creatinine (r = 0.255, p < 0.001) showed positive correlations with age (Fig. 3B, C, and 3D). However, urine creatinine levels (P < 0.001) and urine osmotic pressure (P < 0.001) decreased with age and reached the lowest point in the old-old group (Table 1), these age-related changes may have influenced the GPNMB analysis results. Henceforth, urine creatinine levels and urine osmotic pressure were not used to normalize the urine GPNMB levels in this study.

Fig. 3 illustrates a significant increase after the age of 60 in GPNMB levels of human biofluids. Considering that 60 years in humans corresponds to 18 months in mice [29], this may suggest a potential relationship with RNA-seq analysis from mouse tissues obtained via the GEO dataset, further studies are needed to directly link tissue expression with biofluid levels. Furthermore, women exhibited slightly higher GPNMB levels than men, the result that was also confirmed through multivariate linear regression analysis.

4.GPNMB is a promising aging biomarker and is related to sex, SII, and TBA

Spearman's correlation analysis demonstrated that the biofluid GPNMB levels also showed a significant association with several immune-related factors. The serum GPNMB levels showed a negative correlation with SII (r = −0.126, p = 0.018) and IGF-1 (r = −0.145, p = 0.010). Furthermore, urine GPNMB levels showed a negative correlation with TBA (r = −0.144, p = 0.010) (Table S2). To investigate whether the age-related changes in the biofluid GPNMB levels were affected by other factors, we performed univariate and multivariate linear regression analyses. IGF-1 and TBA significantly change with age (Table 1) and showed a significant correlation with the biofluid GPNMB levels (Table S2). In addition, the previous studies suggested sex [30], BFP [31], SMI [32], SII, and hs-CRP [33,34] may influence biofluid GPNMB levels. These factors were therefore selected to be included in the linear regression analyses.

In the univariate linear regression analysis (Table 2), serum GPNMB levels showed a positive association with age (P = 0.002) and a negative association with SMI (P = 0.046) and IGF-1 (P = 0.002). In the multivariate linear regression analysis (Table 2), serum GPNMB levels showed a significant association with age (β-coefficient = 200.497, 95 % confidence interval [CI] 125.236–275.757, p < 0.001) in model 1 (unadjusted). The correlation between serum GPNMB levels and age remained significant after adjustment for sex in model 2 (β-coefficient = 206.877, 95 % CI 131.124–282.630, p < 0.001), or adjusting for sex, BFP and SMI in model 3 (β-coefficient = 217.498, 95 % CI 133.361–301.635, p < 0.001), or adjusting for sex, BFP, SMI, SII, and hs-CRP in model 4 (β-coefficient = 208.582, 95 % CI 124.222–292.942, p < 0.001). Furthermore, the serum GPNMB levels showed a significant positive correlation with age (β-coefficient = 262.391, 95 % CI 160.994–363.787, p < 0.001), and a negative relationship with SII (β-coefficient = −5.556, 95 % CI −10.721 to −0.391, p = 0.035) after adjusting for sex, BFP, SMI, SII, hs-CRP, and IGF-1 in model 5.

**Table 2:** Univariate and multivariate regression analysis for serum GPNMB levels according to age.

Variables	Univariate analysis				
	Unadjusted coefficient	Standardized coefficient	95 % CI		*p* value
			Lower limit	Upper limit	
Age (years)	70.319	0.141	25.692	114.946	**0.002**
Sex^a^	−858.219	−0.047	−2526.756	810.318	0.313
BFP	133.779	0.083	−49.745	317.303	0.152
SMI	−6899.604	−0.116	−13669.375	−129.833	**0.046**
SII	−3.083	−0.095	−6.484	0.319	0.076
Hs-CRP (mg/L)	−542.976	−0.088	−1222.33	136.378	0.117
IGF-1 (ng/mL)	−31.61	−0.17	−51.948	−11.273	**0.002**
Variables	Multivariate analysis				
	Unadjusted coefficient	Standardized coefficient	95 % CI		*p* value
			Lower limit	Upper limit	
model 1					
Age (years)	200.497	0.299	125.236	275.757	< **0.001**
model 2					
Age (years)	206.877	0.308	131.124	282.630	< **0.001**
Sex^a^	−1469.276	−0.076	−3647.696	709.143	0.185
model 3					
Age (years)	217.498	0.324	133.361	301.635	< **0.001**
Sex^a^	−1999.465	−0.104	−5286.216	1287.285	0.232
BFP	−102.693	−0.063	−403.969	198.582	0.503
SMI	−549.767	−0.009	−14458.489	13358.954	0.938
model 4					
Age (years)	208.582	0.311	124.222	292.942	< **0.001**
Sex^a^	−1622.534	−0.084	−5008.243	1763.176	0.346
BFP	−42.462	−0.026	−351.58	266.655	0.787
SMI	−584.193	−0.01	−14409.031	13240.644	0.934
SII	−5.101	−0.112	−10.267	0.065	0.053
Hs-CRP (mg/L)	−356.902	−0.064	−1032.22	318.416	0.299
model 5					
Age (years)	262.391	0.391	160.994	363.787	< **0.001**
Sex^a^	−1734.583	−0.09	−5107.334	1638.168	0.312
BFP	−85.279	−0.052	−396.326	225.768	0.59
SMI	−2245.79	−0.037	−16120.598	11629.017	0.75
SII	−5.556	−0.122	−10.721	−0.391	**0.035**
Hs-CRP (mg/L)	−231.829	−0.041	−916.995	453.336	0.506
IGF-1 (ng/mL)	23.79	0.135	−1.328	48.909	0.063

BFP, body fat percentage; Hs-CRP, hypersensitive C-reactive protein; IGF-1, insulin-like growth factor 1; SII, systemic immune-inflammation index; SMI, skeletal muscle mass index.

a Sex: Assign 0 to female and 1 to male.

In the univariate linear regression analysis (Table 3), urine GPNMB levels showed a positive association with age (P = 0.022) and a negative association with TBA (P = 0.017). In the multivariate linear regression analysis (Table 3), urine GPNMB levels showed a significant association with age (β-coefficient = 2.970, 95 % CI 1.005–4.935, p = 0.003) in model 1 (unadjusted). The correlation between urine GPNMB levels and age was significant (β-coefficient = 3.331, 95 % CI 1.381–5.282, p < 0.001) after adjusting for sex in model 2. Furthermore, urine GPNMB levels were significantly different between the two sexes. The urine GPNMB levels were lower in men than in women (β-coefficient = −87.022, 95 % CI −142.909 to −31.134, p = 0.002). The correlation between urine GPNMB levels and age remained significant after adjusting for sex, BFP and SMI in model 3 (β-coefficient = 3.967, 95 % CI 1.804–6.130, p < 0.001). The correlation between urine GPNMB levels and sex also remained significant based on model 3 (β-coefficient = −129.624, 95 % CI −214.110 to −45.139, p = 0.003). The urine GPNMB levels showed a positive correlation with age after adjusting for sex, BFP, SMI, SII, and hs-CRP in model 4 (β-coefficient = 3.825, 95 % CI 1.646–6.003, p < 0.001). The differences in urinary GPNMB levels between sexes remained significant in model 4 (β-coefficient = −125.12, 95 % CI −212.466 to −37.774, p = 0.005). The urine GPNMB levels showed a significant positive relationship with age (β-coefficient = 3.871, 95 % CI 1.711–6.031, p < 0.001) and a negative relationship with TBA (β-coefficient = −15.589, 95 % CI −28.250 to −2.928, p = 0.016) after adjusting for sex, BFP, SMI, SII, hs-CRP, and TBA in model 5. Furthermore, differences in urine GPNMB levels between sexes remained significant in model 5 (β-coefficient = −114.412, 95 % CI −201.444 to −27.379, p = 0.010).

**Table 3:** Univariate and multivariate regression analysis for urine GPNMB levels according to age.

Variables	Univariate analysis				
	Unadjusted coefficient	Standardized coefficient	95 % CI		*p* value
			Lower limit	Upper limit	
Age (years)	1.341	0.105	0.192	2.489	**0.022**
Sex^a^	−4.554	−0.01	−47.35	38.242	0.834
BFP	3.37	0.082	−1.299	8.039	0.157
SMI	−162.459	−0.107	−334.837	9.919	0.065
SII	−0.057	−0.069	−0.144	0.03	0.2
Hs-CRP (mg/L)	−10.901	−0.075	−26.866	5.063	0.18
TBA (μmol/L)	−13.779	−0.133	−25.12	−2.437	**0.017**
Variables	Multivariate analysis				
	Unadjusted coefficient	Standardized coefficient	95 % CI		*p* value
			Lower limit	Upper limit	
model 1					
Age (years)	2.97	0.174	1.005	4.935	**0.003**
model 2					
Age (years)	3.331	0.195	1.381	5.282	< **0.001**
Sex^a^	−87.022	−0.177	−142.909	−31.134	**0.002**
model 3					
Age (years)	3.967	0.232	1.804	6.13	< **0.001**
Sex^a^	−129.624	−0.264	−214.11	−45.139	**0.003**
BFP	−0.386	−0.009	−8.169	7.397	0.922
SMI	170.92	0.111	−188.182	530.022	0.35
model 4					
Age (years)	3.825	0.224	1.646	6.003	< **0.001**
Sex^a^	−125.12	−0.255	−212.466	−37.774	**0.005**
BFP	0.602	0.014	−7.42	8.623	0.883
SMI	170.926	0.111	−187.726	529.578	0.349
SII	−0.1	−0.086	−0.234	0.034	0.144
Hs-CRP (mg/L)	−5.302	−0.037	−22.819	12.216	0.552
model 5					
Age (years)	3.871	0.226	1.711	6.031	< **0.001**
Sex^a^	−114.412	−0.233	−201.444	−27.379	**0.01**
BFP	0.093	0.002	−7.87	8.057	0.982
SMI	149.62	0.097	−206.376	505.617	0.409
SII	−0.09	−0.077	−0.223	0.043	0.185
Hs-CRP (mg/L)	−5.658	−0.039	−23.028	11.711	0.522
TBA (μmol/L)	−15.589	−0.14	−28.25	−2.928	**0.016**

BFP, body fat percentage; Hs-CRP, hypersensitive C-reactive protein; SII, systemic immune-inflammation index; SMI, skeletal muscle mass index; TBA, total bile acid.

a Sex: Assign 0 to female and 1 to male.

In summary, biofluid GPNMB levels showed a significant association with age in univariate linear regression analysis and all five models used for the multivariate linear regression analysis, thus, GPNMB was a promising aging biomarker. In univariate analyses, SMI and IGF-1 demonstrated significant changes with GPNMB in serum, suggesting their potential role as correlates of GPNMB. Previous studies indicated that SMI is a reliable predictor of functional outcomes and disability in elderly individuals [17], while IGF-1 is essential for neurogenesis in the adult brain and serves as a marker of cognitive decline in normal aging [27]. However, when we accounted for various confounding factors in our multivariate models (Model 1-Model 5), these significant trends of SMI and IGF-1 did not persist. This indicates that the associations of SMI and IGF-1 with serum GPNMB were not as robust as those observed for age and SII. Additionally, age and TBA both showed a significant relationship with urine GPNMB in univariate and multivariate linear regression analyses. The correlation between sex and urine GPNMB was significant after multiple factors were included. Consequently, our discussion focuses on the correlations of age, SII, and TBA with GPNMB.

## 4 Discussion

Previous studies have reported that myokines, adipokines, and osteokines play a key role in aging. The elevated levels of adiponectin (adipokine) [35], TNF-α (adipo-myokine) [36], and IL-6 (adipo-myokine) [37], and a decline in the levels of leptin (adipokine) [38] and BDNF (myokine) [39] are associated with aging. In this study, we analyzed the relationship between expression levels of myokines, adipokines, and osteokines with age using the bulk RNA-sequencing dataset from mice in the GEO database. Our data demonstrated a significant association between the expression levels of the osteokine GPNMB and aging in mice. This suggested that GPNMB was a potential biomarker of aging. Therefore, we conducted a cross-sectional study of an aging Chinese population to evaluate the association between aging and GPNMB levels in biofluids such as blood and urine in humans.

Firstly, the baseline characteristics of our study population were consistent with those reported in previous studies [40,41]. Aging is manifest as a time-related deterioration of physiological functions, including changes in the body composition such as loss of bone and muscle mass, muscle strength, and subcutaneous fat, and gain of abdominal fat; systemic changes such as altered hormone and blood lipid levels, and blood pressure; mechanical and structural changes such as vascular stiffness that affects heart and brain functions; and altered responses of tissues to hormones, including insulin resistance [40]. In this cohort, all of these parameters showed age-related changes (Table 1), and the participants were healthy and the levels of the baseline characteristics were within the normal range in the four groups, further proving that our cohort is an effective aging cohort. However, the NLR, PLR, LMR, and SII are used as inflammatory biomarkers to assess systemic inflammatory response [42,43]. In our study, the LMR and PLR values were higher in the young and middle-aged groups, but the corresponding blood counts were within the normal range. This suggested that the higher values for the LMR and PLR were caused by stress-related inflammation in younger subjects [44,45].

The serum and urine GPNMB levels both demonstrated age-related correlations in correlation analyses, as well as in univariate and multivariate regression analyses. These findings suggest that biofluid GPNMB levels are promising biomarkers for aging. However, we also detected age-related changes in the urine creatinine levels and urine osmotic pressure. These changes were consistent with previously published reports [46,47]. Since the normalization of urinary creatinine/osmotic pressure may cause an erroneous estimation of the urine biomarkers, we did not normalize the urine GPNMB concentration in this study. Furthermore, we observed a significant positive correlation between serum β-galactosidase activity and age (P = 0.012, Table 1), affirming that our cohort is suitable for aging-related research. It is quite interesting that, although both serum β-galactosidase activity and biofluid GPNMB levels were positively correlated with age, serum β-galactosidase activity and serum GPNMB levels exhibited a negative correlation (P = 0.003, Table S2). This could imply a complex regulatory mechanism between these two biomarkers in the aging process. It suggests that while both are upregulated with age, they could be part of different pathways or processes that counterbalance each other in some way. Previous research has indicated that GPNMB functions as a survival factor in senescent cells; knocking down GPNMB in human vascular endothelial cells was found to shorten their replicative lifespan and increase the activity of senescence-associated β-galactosidase [15]. Our findings further suggest that GPNMB could serve as a potential ageing biomarker.

GPNMB played a key role in the neuroprotective and anti-inflammatory functions. The expression levels of GPNMB are elevated in the substantia nigra of patients with Parkinson's disease (PD) and the GFAP-positive astrocytes [48]. In the astrocyte cell lines and primary mouse astrocytes, GPNMB suppressed cytokine-induced elevation of the levels of inducible nitric oxide synthase, nitric oxide, reactive oxygen species, and IL-6 [48]. Budge et al. reported that the transgenic overexpression of *GPNMB* reduced gliosis, prevented morphological changes in the microglia, and protected against dopaminergic neurodegeneration in the PD mouse model [49]. Furthermore, recombinant GPNMB attenuated lipopolysaccharide (LPS)-induced inflammation in the primary murine microglia [49]. In autoimmune diseases, GPNMB has anti-inflammatory properties, primarily exhibited in macrophages, where it suppresses the production of pro-inflammatory cytokines such as IL-6 and IL-12p40 [50]. Additionally, in psoriatic arthritis, GPNMB is highly upregulated in synovial membranes and peripheral blood cells, suggesting its potential as a biomarker [50]. Furthermore, the interaction between GPNMB on antigen-presenting cells and syndecan-4 on T cells inhibits T-cell activation, which is relevant in T cell-driven autoimmune diseases [50].

GPNMB delayed metabolic damage caused by the induction of obesity and played an immune‐balancing function [51]. High-fat diet-fed *Gpnmb* knockout mice developed more significant metabolic disorders, including insulin resistance, adipose tissue inflammation, and liver fibrosis [51,52]. GPNMB inhibited the polarization of macrophages into the pro-inflammatory phenotype, thereby ameliorating adipose tissue inflammation in obese individuals [52]. Mechanistically, GPNMB performed anti-inflammatory functions by inhibiting macrophage infiltration and the T-cell immune responses through CD44 binding [48,52,53]. This suggested that the elevated levels of GPNMB may protect against aging by regulating inflammation.

GPNMB is also related to tissue repair and regeneration, and shows immense potential in the treatment of bone defects, muscle atrophy, kidney injury, and liver damage [53]. Macrophage-derived GPNMB promoted migration and proliferation of mesenchymal stem cells (MSCs) by engaging with CD44 as a target receptor [54]. Macrophage-associated GPNMB also potentiated tissue reparative M2 macrophage response [54]. This suggested that an increase in the GPNMB levels was necessary for the homeostatic physiological functions. Therefore, deficiency in GPNMB may adversely affect physiological homeostasis and promote disease in the human body.

In various types of cancer, the overexpression of GPNMB promoted the migration, invasion, and metastasis of tumor cells [12,13]. Because of the properties of high GPNMB expression at the surface of cancer cells but significant expression intracellularly in normal cells, GPNMB-targeting monoclonal antibodies are promising candidates for the treatment of patients with GPNMB-expressing cancers [13]. Conversely, GPNMB can exhibit tumor-suppressive effects in certain contexts. For instance, in prostate cancer, GPNMB significantly inhibits cell proliferation and invasion by enhancing the expression of Ndrg1 and maspin genes [13]. These findings suggest that GPNMB has diverse biological functions due to its complex environmental dependency. Aging and cancer are highly correlated biological phenomena [55], often accompanied by chronic inflammation. Consequently, GPNMB tends to be upregulated in both aging and cancer, which could be a response to the inflammatory microenvironment, the underlying mechanism needs to be further studied.

Our results demonstrated that serum GPNMB levels were positively correlated with age and negatively associated with SII. Previous studies have shown that SII is an inflammation-based biomarker associated with several clinicopathological parameters and is an effective prognostic factor in inflammation-related diseases [18]. Inflammation is associated with many chronic diseases among the elderly, including diabetes, cardiovascular diseases, and cancer [56]. SII is associated with aging-related diseases, including sarcopenia [57], dementia [58], and cancer [59]. Elevated SII levels are associated with an increased prevalence of sarcopenia in middle-aged and elderly adults [57], increased risk of cancer development [59], and all-cause dementia [58]. In our study, multivariate linear regression analysis showed a negative correlation between SII and serum GPNMB levels (Table 2), but there was no significant correlation between SII and age (Table 1). This showed that the progressive increase in the serum GPNMB levels may be necessary to maintain a stable SII state during aging and may represent a negative feedback mechanism that regulates the inflammatory response of the human body. Therefore, GPNMB may play a protective role during aging.

Multivariate linear regression analysis also showed gender differences in the urine GPNMB levels, with men showing lower urine GPNMB levels compared to the women (Fig. 3 and Table 3). This may be linked to the sex-related differences in the kidney [60], urinary proteome [30], and urinary tract biology and physiology [61]. Moreover, chronic inflammation is an important biological process that is associated with aging and age-related diseases, and occurs with varying frequencies among men and women [62]. The impact of inflammation caused by stress and insufficient sleep is higher in women than in men [62]. Age-related changes in the immune system are significantly different between men and women. The T cell defense mechanisms are weaker and the pro-inflammatory responses are stronger among women [63]. Since GPNMB is associated with anti-inflammatory effects [33], it is plausible that the higher GPNMB levels may alleviate inflammation-associated injury in females [62].

Multivariate linear regression analysis also showed higher urine GPNMB levels correlated with lower TBA levels (Table 3). TBA is a biomarker of liver function and is an indicator of hepatobiliary injury or disease [64]. GPNMB plays a key role in the liver. In the mouse model of acute liver injury, *Gpnmb*-positive macrophages infiltrated the liver and contributed to the balance between fibrosis and fibrolysis during the repair process [65]. Therefore, the results of our study suggested that elevated GPNMB levels may be part of the anti-inflammatory and anti-apoptotic mechanisms that are required for the regulation of normal liver function. Moreover, lower TBA levels are associated with normal liver function.

This study has a few limitations. The sample size was relatively small, which limits the generalizability of our findings. Larger cohort studies are necessary to confirm our results and minimize the impact of individual differences. Additionally, further validation through longitudinal studies is required to strengthen our conclusions. To address this, we plan to conduct follow-up investigations with the current participants to further explore the potential of GPNMB as an aging biomarker. The aging process and age-related diseases are complex and are not mediated by a single protein, but rather by highly interconnected networks of pathways involving multiple proteins [66,67]. Moreover, various genetic and environmental factors also influence the aging process and need to be considered in the future. Therefore, further investigations are necessary to fully elucidate the functions of GPNMB in aging using both *in vitro* and *in vivo* models.

## 5 Conclusions

In summary, this study demonstrated that the biofluid GPNMB was a strong clinical biomarker candidate for estimating biological aging.

## Ethics approval and consent to participate

The study protocol was approved by the Research Ethics Committee of Beijing Hospital (2019BJYYEC-054-02), and informed consent was obtained from all participants. The study was performed in accordance with the principles of the Declaration of Helsinki.

## Consent for publication

Not applicable.

## Funding

This work was supported by the 10.13039/501100001809National Natural Science Foundation of China (82073264, 81770228, 81970745); National High Level Hospital Clinical Research Funding (BJ-2024-219); the 10.13039/501100012166National Key Research and Development Program of China (2020YFC2002700, 2018YFC2000300); the CAMS Innovation Fund for Medical Sciences (No. 2021-I2M-1–050); 10.13039/100019176Shenzhen Institute of Synthetic Biology Scientific Research Program (DWKF20210009).

## Data availability statement

A readily available data set (GSE132040) was downloaded from the Gene Expression Omnibus (GEO) at the NCBI (https://www.ncbi.nlm.nih.gov/geo/). Other data used and/or analyzed during the current study are available from the corresponding author on reasonable request.

## CRediT authorship contribution statement

**Yuan-Yuan Liu:** Writing – review & editing, Writing – original draft, Visualization, Validation, Software, Methodology, Investigation, Formal analysis, Data curation. **Jing Pang:** Writing – review & editing, Writing – original draft, Methodology, Funding acquisition, Data curation. **Chi Zhang:** Visualization, Validation, Formal analysis, Data curation. **Lv-Tao Zeng:** Visualization, Software, Methodology, Formal analysis, Data curation. **Yao Wang:** Methodology, Investigation, Data curation. **Shi-Bo Wang:** Investigation. **Guo-Qing Fan:** Validation, Software, Methodology, Formal analysis, Data curation. **Li-Qun Zhang:** Formal analysis, Data curation. **Tao Shen:** Writing – review & editing, Funding acquisition. **Xue-Fei Li:** Software, Methodology, Formal analysis. **Chuan-Bao Li:** Validation, Methodology, Data curation. **Su-Yan Cao:** Data curation. **Tie-Mei Zhang:** Supervision, Project administration, Conceptualization. **Jian-Ping Cai:** Supervision, Project administration, Funding acquisition, Conceptualization. **Ju Cui:** Writing – review & editing, Writing – original draft, Supervision, Project administration, Methodology, Funding acquisition, Data curation, Conceptualization.

## Declaration of competing interest

The authors declare that they have no known competing financial interests or personal relationships that could have appeared to influence the work reported in this paper.

## List of abbreviations

ALT: alanine aminotransferase
ANOVA: analysis of variance
AST: aspartate aminotransferase
BASO %: basophil percentage
BAT: brown adipose tissue
BIA: bioelectrical impedance analysis
BFP: body fat percentage
BMI: body mass index
CALS: China Ageing Longitudinal Study
CI: confidence interval
CRE: blood creatinine
ELISA: enzyme-linked immunosorbent assay
EO %: eosinophil percentage
FAS-eGFR: full age spectrum-estimated glomerular filtration rate
FBG: fasting blood glucose
GAT: gonadal adipose tissue
GEO: Gene Expression Omnibus
GGT: glutamyl transpeptidase
GPNMB: glycoprotein non-metastatic melanoma B
Hb: hemoglobin
HDL-C,: high density lipoprotein-C
Hs-CRP: hypersensitive C-reactive protein
IGF-1: insulin-like growth factor 1
IL,: interleukin
LDL-C,: low density lipoprotein-C
LMR: lymphocyte-to-monocyte ratio
LPS: lipopolysaccharide
MAT: mesenteric adipose tissue
MPTP: 1-methyl-4-phenyl-1,2,3,6-tetrahydropridine
MSC: mesenchymal stem cell
NEFA: non-esterified fatty acid
NF-κB: nuclear factor kappa B
NLR: neutrophil-to-lymphocyte ratio
PD: Parkinson's disease
PLR: platelet-to-lymphocyte ratio
RBC: red blood cell
SCAT: subcutaneous adipose tissue
SD: standard deviation
SII: systemic immune-inflammation index
SMI: skeletal muscle mass index
TBA: total bile acid
TC: total cholesterol
TG: triglyceride
TNF: tumor necrosis factor
UREA: blood urea
URIC: blood uric acid
WBC: white blood cell
